# Transcriptomic and Mutational Analysis Discovering Distinct Molecular Characteristics Among Chinese Thymic Epithelial Tumor Patients

**DOI:** 10.3389/fonc.2021.647512

**Published:** 2021-09-08

**Authors:** Naixin Liang, Lei Liu, Cheng Huang, Hongsheng Liu, Chao Guo, Ji Li, Weiwei Wang, Nan Li, Rui Lin, Tao Wang, Lieming Ding, Li Mao, Shanqing Li

**Affiliations:** ^1^Department of Thoracic Surgery, Peking Union Medical College Hospital, Chinese Academy of Medical Sciences, Beijing, China; ^2^Department of Pathology, Peking Union Medical College Hospital, Chinese Academy of Medical Sciences, Beijing, China; ^3^Department of R&D, Hangzhou Repugene Technology Co., Ltd., Hangzhou, China; ^4^Department of Medical, Betta Pharmaceuticals Co., Ltd., Hangzhou, China

**Keywords:** thymic epithelial tumor, thymoma, next-generation sequencing, PD-L1, RNA-seq

## Abstract

**Introduction:**

Thymic epithelial tumors (TETs) are malignancies arising from the epithelium of the thymic gland, rare but with relatively favorable prognosis. TETs have different pathological subtypes: thymomas and thymic carcinoma, and they show different clinical characteristics regarding prognosis, pathology, and molecular profiles, etc. Although some studies have investigated the pathogenesis of TETs, more molecular data is still needed to further understand the underlying mechanisms among different TETs subtypes and populations.

**Methods:**

In this study, we performed targeted gene panel sequencing and whole transcriptome sequencing on the tumor tissues from 27 Chinese TET patients, including 24 thymomas (A, AB, and B subtypes) and 3 thymic squamous cell carcinomas. We analyzed the genetic variations and differentially expressed genes among multiple TET subtypes. Moreover, we compared our data with the published The Cancer Genome Atlas (TCGA) TET data on both the genetic and transcriptomic levels.

**Results:**

Compared with the TCGA TET genomic data, we found that *NF1* and *ATM* were the most frequently mutated genes (each with a frequency of 11%, 3/27). These mutations were not mutually exclusive, since one B1 thymoma showed mutations of both genes. The *GTF2I* mutation was mainly enriched in subtype A and AB thymomas, consistent with the previous reports. RNA-seq results unveiled that the genes related to thymus development (*FGF7*, *FGF10* and *CLDN4*) were highly expressed in certain TET subtypes, implicating that the developmental process of thymus might be linked to the tumorigenesis of these subtypes. We found high expression of *CD274* (PD-L1) in B2 and B3 thymoma samples, and validated its expression using immunohistochemistry (IHC). Based on the expression profiles, we further established a machine learning model to predict the myasthenia gravis status of TET patients and achieved 90% sensitivity and 70.6% specificity in the testing cohort.

**Conclusion:**

This study provides the first genomic and transcriptomic analysis of a Chinese TET cohort. The high expression of genes involved in thymus developmental processes suggests the potential association between tumorigenesis of TETs and dysregulation of developmental pathways. The high expression of PD-L1 in B2 and B3 thymomas support the potential application of immunotherapy on certain thymoma subtypes.

## 1 Introduction

Thymoma and thymic carcinoma (TC) are thymic epithelial tumors (TETs) with low occurrence rate, roughly 1-5 cases per million population per year (1, 2). Pathologically, thymomas can be stratified into A, AB, B1, B2 and B3 subtypes depending on the morphology and the proportion of cancer cells and lymphocytes (3). A and AB thymomas are generally considered to be low malignancy, whereas B and TC subtypes are associated with moderate and high malignancy, respectively (1, 2). Auto-immune disorders, such as myasthenia gravis (MG), are the most frequent syndrome co-occurring with thymomas (4). A few studies have focused on characterizing genomic variations and expression of certain genes in thymomas and TCs (5–9), which provide new insights to decipher mechanisms of tumorigenesis and develop novel therapeutic strategies for clinical practice. However, more molecular data is still needed to deeply understand the TET etiology among different subtypes and populations.

Mutations and aberrant expression levels of several genes have been identified in thymoma and TC. *EGFR* is highly expressed in some thymoma and TC samples, but only a few mutations have been identified within *EGFR* in thymoma samples (10). Mutations and overexpression of *ERBB2*, *KRAS*, and *TP53* are found in TC samples (5). The high expression of *KIT* has also been confirmed in TCs (10, 11). However, mutations have been rarely found in *KIT* in either TCs or thymomas (12). A leucine to histidine substitution (L383H, L404H) of *GTF2I* was recently identified to be one of the most frequent mutations in A and AB thymomas (13). *In vitro* experiments showed the mutations were associated with the tumorigenesis of thymomas (13). Radovich et al. also demonstrated the high prevalence of GTF2I L424H mutation in A and AB subtypes (6).

The application of next-generation sequencing (NGS) has greatly broadened the mutational landscape of thymomas and TCs. Wang et al. identified mutations of several genes related to epigenetic regulation in both thymomas and TCs, including *BAP1*, *SETD2*, *ASXL1*, *SMARCA4*, *DNMT3A*, *TET2*, and *WT1* (8). Mutations of the RAS family genes, including *HRAS* and *NRAS*, were also identified by a 50-gene panel on A, B3, and TC subtypes (7). Another study identified recurrent somatic mutations of *TET2*, *CYLD*, *SETD2*, *TP53*, *FBXW7*, *HRAS*, and *RB1*genes on TC samples by whole exome sequencing (WES) (9). These studies provide a more thorough understanding of the mutational landscapes of TET, although mechanistic insight is still needed to understand the relationship between different subtypes and to provide new clues for development of therapeutic strategies.

The TCGA TET study represents the most systematic investigation on molecular profiles of thymomas and TCs so far (6). In the study, 117 samples from various thymoma subtypes and TCs were analyzed by WES, RNA-seq, miRNA-seq, DNA methylation and RPPA arrays. Unsupervised clustering resulted in four clusters which were consistent with the pathologic classifications. The auto-immune MG was linked to somatic copy number variations and the intratumor over-expression of auto-antigen related genes, such as *CHRNA1*, *NEFM*, and *RYR3* (6). *GTF2I* mutated samples had higher expression in several pathways related to cancer and cell signaling. Meanwhile, the TCGA TET study still leaves the interpretation of expression differences between subtypes as an open question. Importantly, it was worth noting that more than 80% of people in the TCGA cohort were Caucasian, and the Asian population was under-represented. More molecular studies on TET patients of other ethnicities are still needed.

In this pilot study, we present a comprehensive analysis on genomic and transcriptomic data of a Chinese TET cohort of 27 patients. To unveil the specific mechanisms involved in TET tumorigenesis of Asians, we made a thorough comparison between our cohort and the TCGA TET cohort on both the genomic variation and expression profiles of each subtypes.

## 2 Materials and Methods

### 2.1 Patients and Sample Collection

Twenty-seven TET patients, including 24 thymomas and 3 thymic squamous cell carcinomas, were enrolled in this study (**Table 1** and **Supplementary Tables 1**, **2**). All patients were Chinese and were treated in Peking Union Medical College Hospital. Clinical characteristic information regarding age, gender, race, histological classification, and clinical stage (Masaoka and TNM staging) were collected. Fresh frozen tumor tissue and white blood cells were collected from each patient during surgery or biopsy with informed consent forms and approval from the Ethics Committee of the Peking Union Medical College Hospital. Part of fresh tissue sampled from multiple distinct regions of the resected tumor tissue was made to one or several formalin-fixed, paraffin-embedded blocks in the pathology department of the hospital. The histologic subtypes for the 27 patients were examined following the 2015 World Health Organization (WHO) classification of tumors of the thymus (4th edition) (14). The CT images and immunohistochemistry (IHC) results were provided in the **Supplementary Tables 1**, **2**. Typical hematoxylin and eosin (H&E) result of each histological subtype (A, AB, B1, B2, B3 and TC) was also obtained (**Supplementary Figure 1**). The remained fresh tissue was frozen by liquid nitrogen and transported to the molecular lab for DNA panel and RNA-seq analysis.

**Table 1:** Clinical characteristics of the 27 thymic epithelial tumor (TET) patients.

Characteristic	All (n=27)
**Age**	
Median	53
Range	25-70
**Sex**	
Male	12 (44%)
Female	15 (56%)
**Race**	
Asian	27 (100%)
**Histologic Type**	
A	2 (7.4%)
AB	6 (22.2%)
B1	4 (14.8%)
B2	7 (25.9%)
B3	5 (18.5%)
TC	3 (11.1%)
**Masaoka Stage**	
I	13 (48.2%)
II	5 (18.5%)
III	9 (33.3%)
**TNM Stage**	
I	13 (48.2%)
II	5 (18.5%)
IIIA	8 (29.6%)
IIIB	1 (3.7%)

### 2.2 Sample Processing

Before DNA and RNA extraction, a frozen tissue section for each sample was cut by a cryostat (Leica CM 1950, Leica Biosystems, Wetzlar, Germany), then fixed on glass slide and stained by Hematoxylin and Eosin (H&E) to examine the tumor percentage of the tissue sample. The tumor cell proportions are confirmed to be above 20%. DNA and total RNA were extracted from fresh frozen tissue using the DNeasy Blood&Tissue Kit (Qiagen, Valencia, CA, USA) and RNeasy Mini Kit (Qiagen), respectively, following the manufacturer’s protocols. Genomic DNA was extracted from peripheral blood using the QIAamp DNA Mini and Blood Mini Kit (Qiagen) per manufacturer’s protocol. The Qubit 3.0 Fluorometer and Qubit dsDNA HS Assay kit (Life Technologies, Carlsbad, CA) were used to quantify DNA following the manufacturer’s recommended protocol. The quality and quantity of extracted RNA were evaluated with NanoDrop 2000 (ThermoFisher, Pittsburgh, PA, USA) and Agilent 2100 Bioanalyzer (Agilent Technologies, Santa Clara, CA, USA).

### 2.3 DNA Sequencing

NGS library preparation was performed to the DNA samples using KAPA Hyper Prep kit (Kapa Biosystems, Wilmington, MA, USA) according to the manufacturer’s instruction and hybridized with probes targeting to the whole exons of 474 cancer-related genes using SureSelectXT Target Enrichment System (Agilent Technologies, Santa Clara, CA, USA). The libraries were sequenced using the HiSeq-X10 platform (Illumina, San Diego, CA, USA).

FASTQ files of raw sequencing reads were generated using bcl2fastq Conversion Software (Illumina, Version: 2.17.1.14). Low quality reads were filtered out and short reads were aligned to hg38 genome using bwa-0.7.15 (15). PCR duplicates were removed using GATK Picard. Indel realignment and base recalibration were performed by GATK to improve indel detection sensitivity and correct bias of base quality scores (16). MuTect2 and GATK were used for single nucleotide variation (SNV) and indel calling, respectively. All variants were annotated with HGVS using snpEff-2.3.7 (17). Only coding region variants (SNV and INDEL) with mutation allele frequencies (MAF) ≥ 5% were retained for further analysis. The tumor mutation burden (TMB) was calculated by counting the non-synonymous somatic mutations in coding regions and normalized by the panel size as previously described (18).

### 2.4 RNA Sequencing

The RNA-seq library was constructed using NEBNext Ultra Directional RNA Library Prep Kit (New England Biolabs, Ipswich, MA, USA) according to the manufacturer’s instructions, and qualified using Qubit 3.0 Fluorometer (Life Technologies, Carlsbad, CA, USA) and 2100 Bioanalyzer (Agilent Technologies, Santa Clara, CA, USA). Libraries were sequenced on HiSeq-X10 platform (Illumina, San Diego, CA, USA).

After FASTQ was generated, low quality reads were filtered out and short reads were mapped to hg38 reference genome and ensemble 93 genome annotation using STAR (19). Gene expression quantification was performed using RSEM (20) to obtain the fragment per kilo exon per million reads (FPKM) value of each gene. Coefficient of variation (CV) was calculated for each gene. Three thousand genes with the largest CV were selected for clustering. All samples were clustered by hierarchical clustering with ward D2 method. Cluster heat maps were generated using pheatmap. The sample and gene cluster numbers were determined using ConsensusCluster (21). Genes with median FPKM larger than 4 were considered as highly expressed in each cluster. STRING web tools was used to perform pathway enrichment analysis of highly expressed genes (22). The differential gene expression analysis among clusters was performed using DESeq2 (23).

### 2.5 Protein-Protein Interaction Network Analysis

PPI network analysis was performed to investigate the potential effects of somatic mutations on cellular functional networks. Mutations in genes were first filtered by the gene expression levels based on the FPKM (fragment per kilo exon per million reads) values (> 4). These highly expressed genes were then mapped to the PPI network from STRING database, and further subjected to gene clustering using the Markov clustering (MCL) method provided on STRING website.

### 2.6 Immunohistochemistry for PD-L1

Before PD-L1 immunohistochemistry (IHC), we first estimated the tumor cell percentage (TCP) of FFPE tissue using hematoxylin and eosin (H&E). FFPE samples with more than 100 tumor cells were further examined by IHC using PD-L1 IHC 22C3 pharmDx (Agilent Technologies, Santa Clara, CA, USA) according to the manufacturer’s instructions. PD-L1 expression in TET tumor tissue is determined by the tumor proportion score (TPS). TPS is the percentage of viable tumor cells showing partial or complete membrane staining at any intensity (≥ 1+) relative to all viable tumor cells present in the sample, which is defined accordingly:

$$\text{TPS }(\%)=\frac{No.\;of\;PD-L1\;staining\;cells\;(tumor\;cells)}{Total\;No.\;of\;viable\;tumor\;cells}\times 100$$

Based on the TPS, the expression level of PD-L1 protein is defined as ‘High expression’ (TPS ≥ 50%), ‘Positive’ (1% ≤ TPS < 50%) and ‘Negative’ (TPS < 1%).

### 2.7 Prediction on Myasthenia Gravis Using SVM

LIB-SVM 3.25 was used for construction of support vector machine (SVM) model in this study (24). Gene expression profiles of the TCGA cohort and our cohort were normalized using the median absolute deviation (MAD) method (6). The prediction power of each gene in the top variable gene list was evaluated using the single gene SVM model by the area under the curve (AUC) from the Receiver Operating Characteristics (ROC) analysis. The forward selection was then used to construct a gene set for prediction. SVM hyper parameters were selected using the top three gene sets. The top three gene set models were used on the validation cohort to evaluate the generalization error performance.

## 3 Results

### 3.1 Clinical Characteristics

Twenty-seven patients with clinical diagnoses of thymoma or TC (thymic squamous cell carcinoma) were enrolled in this study (**Table 1** and **Supplementary Tables 1**, **2**). The median age of all the patients was 53, ranging between 25 and 70 years, and there were 12 male and 15 female patients. The histologic types for the 27 patients were determined by pathological examination following the 2015 World Health Organization (WHO) classification of tumors of the thymus (4th edition) (14), which were classified into A type (n = 2), AB type (n = 6), B1 type (n = 4), B2 type (n = 7), B3 type (n = 5), and TC (n = 3) (**Supplementary Figure 1**). All the three TC samples were histologically diagnosed as thymic squamous cell carcinomas with CD5+ and CD117+. Most of the patients were in Masaoka stage I (n = 13), with the remained patients distributed in stage II (n = 5), and stage III (n = 9). For TNM staging, there were also 13, 5, and 9 patients distributed in stage I, II, and III, respectively.

### 3.2 Genome Variation of Subtypes in TETs

**Figure 1:**
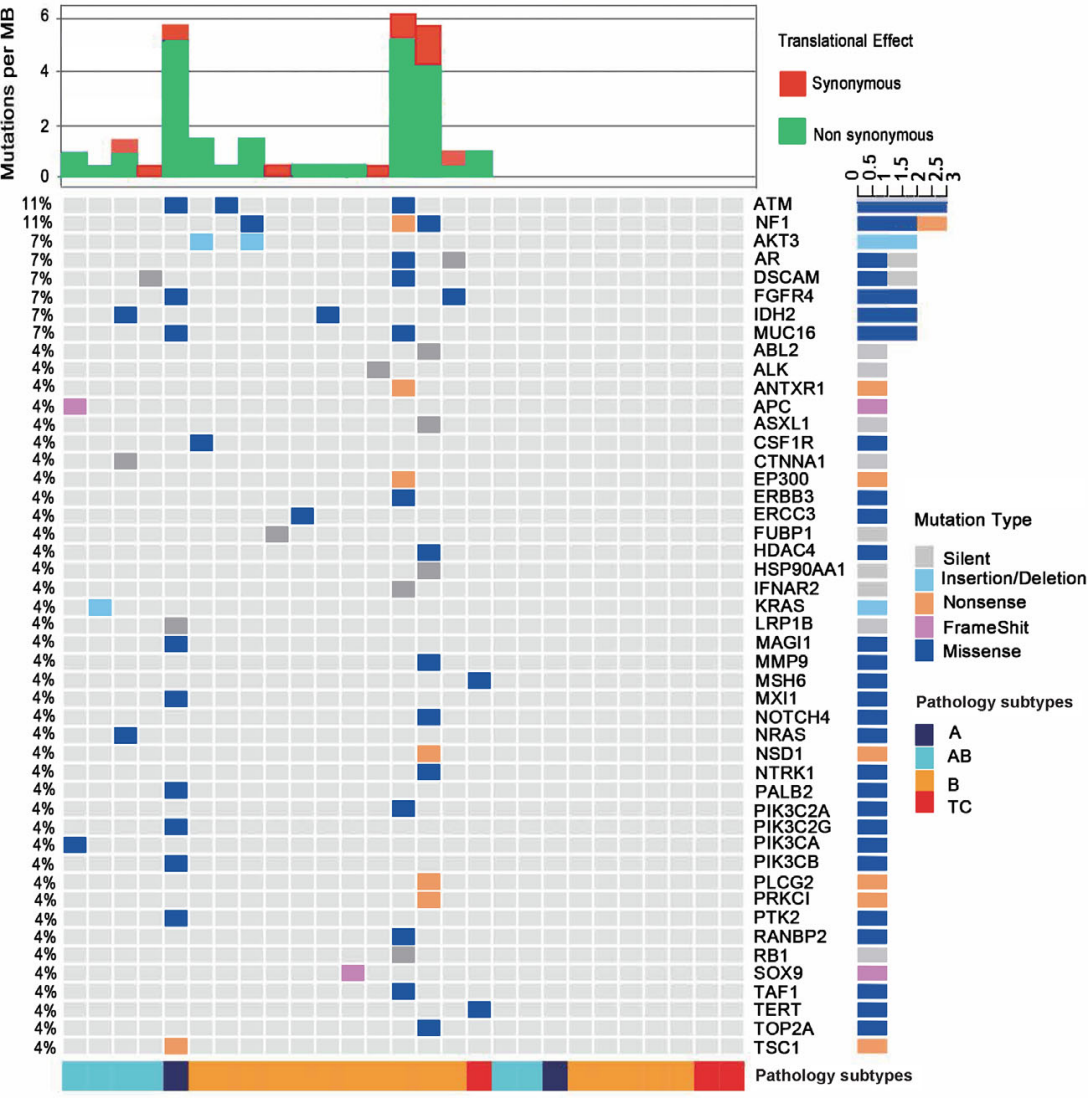
Mutational landscape of the 27 patients with thymoma or thymic carcinoma. The percentages in the left showing the frequency of each gene mutated in the cohort. The bottom of the figure shows pathological types of each sample. Type B contains all B subtypes, including B1, B2 and B3. The top of the figure shows mutational burden for each sample, red for synonymous mutations and blue for non-synonymous mutations.

DNA samples from the paired tissue and white blood cells for each patient were sequenced using a 476-gene panel (**Supplementary Table 3**). In total, 27 tissue samples from thymoma or TC were sequenced. We used MAF ≥ 5% as the cutoff for variant filtering. Fifty-eight genomic variations from 47 genes were identified (**Figure 1**). The average TMB of the 27 samples was as low as 0.82 mut/MB, which was consistent with the low mutation burden discovered in the TCGA TET study (6). Ten samples possess a TMB of 0 mut/MB. Four of the six samples with TMB > 1 mut/MB were found in the B subtypes. One A subtype and two B subtypes had exceptionally high TMB > 5 muts/MB. Of the three TC samples, only the sample with MSH6 mutation had a TMB of 0.95 mut/MB and the other two was 0 mut/MB. Consistently, the two carcinomas from Asian patients in the TCGA cohort also had a low mutational burden (0.13 and 0.66 mut/MB).

Among the 47 genes carrying at least one mutation, *NF1* and *ATM* were the most frequently mutated genes (11%, 3/27) in all samples. The predicted pathogenicity of the identified mutations was also retrieved from NCBI ClinVar database (25). Three *NF1* mutations were detected in one B1 (G1090*, nonsense mutation, unknown), one B2 (D1067V, missense mutation, likely benign) and one B3 (P1087L, missense mutation, unknown) subtypes. Three *ATM* mutations were identified in one A (P424H, missense mutation, uncertain significance), one B1 (R493G, missense mutation, unknown) and one B3 (S169F, missense mutation, uncertain significance) subtypes. These mutations were not mutually exclusive, since the B1 thymoma showed mutations of both genes. One AB subtype had a *KRAS* A59del mutation, and another AB subtype had an *NRAS* Q61K mutation. Only one TC sample were identified to have somatic mutations, which are MSH6 I927M (missense mutation, uncertain significance) and TERT R972S (missense mutation, unknown). IHC result didn’t show loss of MSH6 protein expression and thus didn’t imply microsatellite instable status. MSH6 I927M is predicted to be uncertain significance as also supported by the finding that the respective TC sample did not exhibit a high TMB as would be expected in a microsatellite instable tumor. The TC samples in our study had fewer somatic mutations than thymoma samples, which might implicate that somatic mutation related tumorigenesis differs in thymomas and TCs. We found significant enrichment of mutated genes in both the RAS (q=7E-8) and PI3K-Akt signaling pathways (q=2E-7), including *AKT3*, *CSF1R*, *FGFR4*, *KRAS*, *NRAS*, *PIK3CA*, and *PIK3CB* (**Figure 1** and **Supplementary Table 4**), and those mutated genes were mainly enriched in thymoma samples, suggesting that RAS and PI3K-Akt signaling pathways may involve in the tumor development of thymoma. In summary, the two most frequently mutated genes in our cohort, *NF1* and *ATM*, could be somatic mutations specific to Chinese TET patients, as no *NF1* or *ATM* mutations were found in the TCGA TET cohort.

### 3.3 Perturbation of Somatic Mutations on Protein-Protein Interactions

The functions of somatic mutations in our TET cohort were further explored for their potential perturbation on cellular PPI network. Twenty-eight highly expressed genes harboring 37 non-synonymous mutations were used for perturbation analysis on the PPI network (**Figure S2A**). Another twenty-two genes were also included in the network if they had at least one interaction with one of the 28 highly expressed genes. Clustering analysis of the 28 genes resulted in 6 clusters on the PPI network. The largest cluster had 9 genes, primarily belonging to the RAS signaling pathway. Other clusters were much smaller and showed no significant function enrichment. Furthermore, we performed the same analysis using the mutational landscape of each subtype separately. Different subtypes showed significantly different network topologies. The largest cluster of A and AB type (**Figures S2B, C**) contained genes in the RAS and PI3K-Akt signaling pathways, but no overlap was observed between the two subtypes. The largest cluster of B subtypes contained four genes that did not show significant enrichment in any pathways (**Figure S2D**). The results from the pathway analyses and PPI network clustering analysis were largely consistent, and suggested that the functional consequences of mutated genes in thymoma were closely related to key signaling pathways in cancer, which may contribute to the tumorigenesis of TET.

### 3.4 *GTF2I* Mutation in Thymoma Samples

The *GTF2I* L424H mutation was a newly identified recurrent genomic variation in A and AB subtypes of thymomas (13) Since the gene was not covered by our gene panel, we analyzed the RNA-seq data for the mutation status of *GTF2I* in our cohort (**Supplementary Table 5**). Eleven samples showed the *GTF2I* L424H mutation, including all the six patients in the AB subtype and one patient in A subtype, which was consistent with the observation in the TCGA-TET study that the *GTF2I* mutation mainly occurred in the A and AB subtypes (6). Besides that, we also detected *GTF2I* L424H mutation in two B2 and two B3 subtypes. The frequency of *GTF2I* mutation in subtype B of our cohort was 25% (4/16), which was roughly equal to the frequency reported by Petrini et al. (24%, 29/122), and higher than that in TCGA cohort (11%, 6/55) (P=0.024). To further validate the *GTF2I* mutation status, we performed Sanger sequencing on three representative samples [i.e., P1 (A), P21 (B3) and P24 (B3)] (**Figure S3**). The Sanger results were consistent with the RNA-seq data, which further confirmed the occurrence of *GTF2I* mutation in thymoma samples.

### 3.5 Hierarchical Clustering on Expression Profiles of Thymomas and Thymic Carcinomas

**Figure 2:**
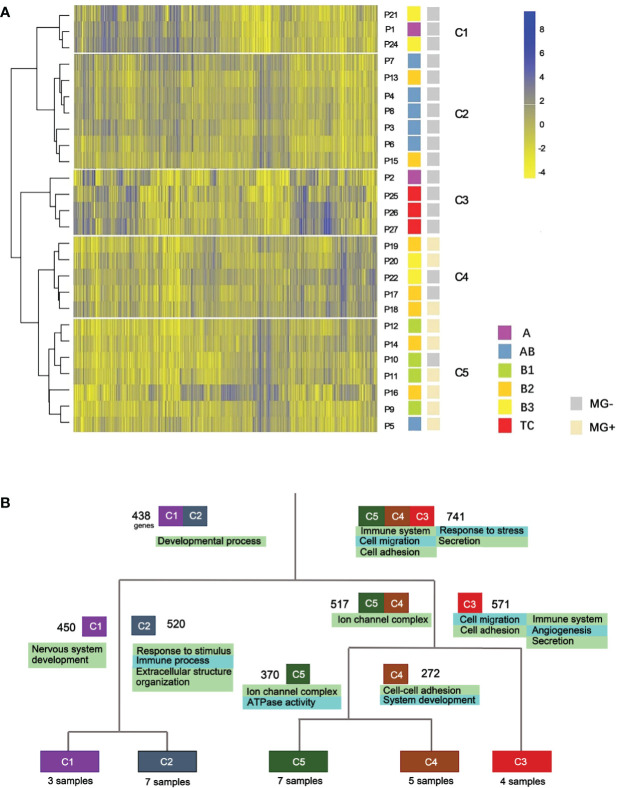
Expression profile clustering of thymoma and thymic carcinoma. **(A)** Five clusters were established. C1-C5, different clusters; P, patient; MG, myasthenia gravis. **(B)** Gene function differences between thymic epithelial tumor types. Cluster-based differential expression analysis was performed. Four splits were used to separate the five clusters. Numbers besides the cluster boxes represent numbers of highly expressed genes.

RNA-seq data from 26 samples passed the quality control and were used for the hierarchical clustering analysis. Based on the distinct expression patterns, 5 clusters (C1-C5) were generated which were closely related to the pathogenic subtypes of thymomas and TCs (**Figure 2A**). Cluster 1 (C1) contains three samples, including one A and two B3 subtypes. Cluster 2 (C2) is an AB dominant cluster that includes five AB and two B2 subtypes. Except for one A subtype, all the other C3 samples are TC subtypes. Clusters C4 and C5 are dominated by B subtypes. Among them, C4 has three B2 and two B3 samples, and C5 includes four B1, two B2 and one AB subtypes. Overall, the expression profiling based clusters was generally consistent with histologic classifications.

We also performed an integrative clustering analysis using a combination of the RNA-seq data from TCGA and our cohort to validate the consistency of the clusters and histologic subtypes. The combined clustering results showed that all C1 members were clustered with either A or AB samples in the TCGA data set. The A subtype that clustered together with TC subtypes, was confirmed to be an outlier in the integrative analysis of TCGA data and our data (**Figure S4**). It is worth noting that the samples from Asian patients of the two cohorts were evenly distributed among non-Asian clusters. No ethnics-specific gene expression profiles were discovered in this analysis.

### 3.6 Expression Differences Among Thymic Epithelial Tumor Subtypes

We next performed cluster-based differential expression analysis to explore the gene functional differences among different TET subtypes. Differential expression analysis was performed within the five clusters of the hierarchical clustering. Differentially expressed genes (DEGs) from each cluster were then analyzed for functional pathways enrichment (**Figure 2B**). We observed that subtypes of TET (C3, C4 and C5) tended to enrich in the pathways related to immune system and cell adhesion/migration, whereas the subtypes of C1 and C2 were enriched in the pathways related to developmental processes and cellular components. In the sub-branches of C1 and C2, the enriched genes functions of the C2 cluster were immune processes, whereas the C1 was related to nervous system development. Those results showed that the differentially expressed genes were more enriched in immune system and cell adhesion/migration pathways in the more malignant subtypes than that in the less malignant subtypes.

**Figure 3:**
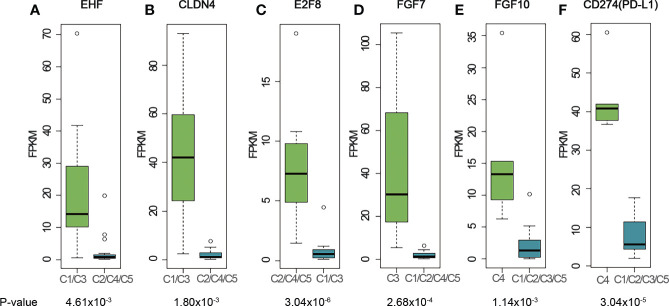
Representative genes highly expressed in different clusters. **(A)** EHF; **(B)** CLDN4; **(C)** E2F8; **(D)** FGF7; **(E)** FGF10; **(F)** CD274 (PD-L1). The cluster name on X-axis uses the histologic type corresponding to the expression profiling cluster. The matches of dominant pathological subtype and RNA-seq clusters are: A, B3 subtypes - C1, AB subtypes - C2, TC subtypes - C3, B2, B3 subtypes - C4, and B1, B2 subtypes - C5. P-values were calculated using Mann-Whitney-Wilcoxon test. FPKM stands for fragment per kilo exon per million reads.

We further sought to identify genes that were specifically expressed in each cluster and analyze their biological functions (**Figure 3A**). Transcription factor *EHF* was highly expressed in C1 (**Figures 3A** and **S5A**). The high expression of *EHF* has been related to the progression of gastric cancer and to the elevation of HER family proteins *ERBB3* and *ERBB4* (26). In our study, C1 samples had higher expression levels of *ERBB3* and *ERBB4* (**Figures S6A, B**), which together with *EHF*, suggested possible implication of the C1 cluster to the development of epithelial malignancies (27–29). Two thymus development related genes, *CLDN4* (**Figures 3B** and **S5B**) and *TNFRSF11A* (**Figure S7B**), were also highly expressed in C1 samples. RANK (coded by *TNFRSF11A*) is highly expressed in medullary progenitor cells and also an important regulator in medullary formation by promoting the generation of AIRE+ mature medullary epithelial cells (30) Claudin-4, which is encoded by *CLDN4*, is a highly expressed gene marker for medullary epithelial stem cells (31). These findings may implicate that the tumorigenesis of A subtype is associated with the deregulation of gene expression and reconstitution of stem cell like properties of medullary epithelial cells.

E2F8 gene, which involves in cell proliferation and cancer development, was found to be highly expressed in C2, C4 and C5 clusters (**Figures 3C** and **S5C**). E2F8 gene was previously reported highly expressed in several different cancers compared to their normal tissues (32, 33). To investigate whether high expression of E2F8 gene was associated with epithelial cells and immature T cells, we calculated the correlation coefficient between E2F8 and TdT gene expressions using RNA-seq data for all the C2, C4 and C5 samples. The result showed a low R-squared value of 0.36 which indicated E2F8 expression was not highly correlated with TdT expression from immature T cells, and thus implied high expression of E2F8 possibly originate from both the immature T cells and epithelial cells. Compared to C1 cluster, we did not find any expression of genes related to development of either medullary or cortex epithelial cells in C2 cluster, which might indicate a difference in tumorigenesis for the AB subtype of thymoma.

C3 contains mostly TC subtypes. *KIT* was highly expressed in all TC subtypes in our cohort (**Figure S8A**), consistent with the previous research (12) We also found that the cell surface receptor *PDGFRA* was highly expressed in the TC subtypes (**Figure S8B**). Although no statistical significance was observed for either *KIT* or *PDGFRA* due to the small size of TC samples, the trend was observed for the median expression level. Several FGF family genes, like *FGF1*, *FGF7* and *FGF11* were also highly expressed in the TC group (*FGF*7, **Figures 3D** and **S5D**; *FGF1*, **Figure S6C**; *FGF11*, **Figure S6D**). In the cortex, mesenchymal cells produce *FGF7* that promotes the proliferation of epithelial cells (34). The high expression of *FGF7* in TC subtypes supports this mechanism of self-maintenance of epithelial proliferation in TC subtypes.

**Figure 4:**
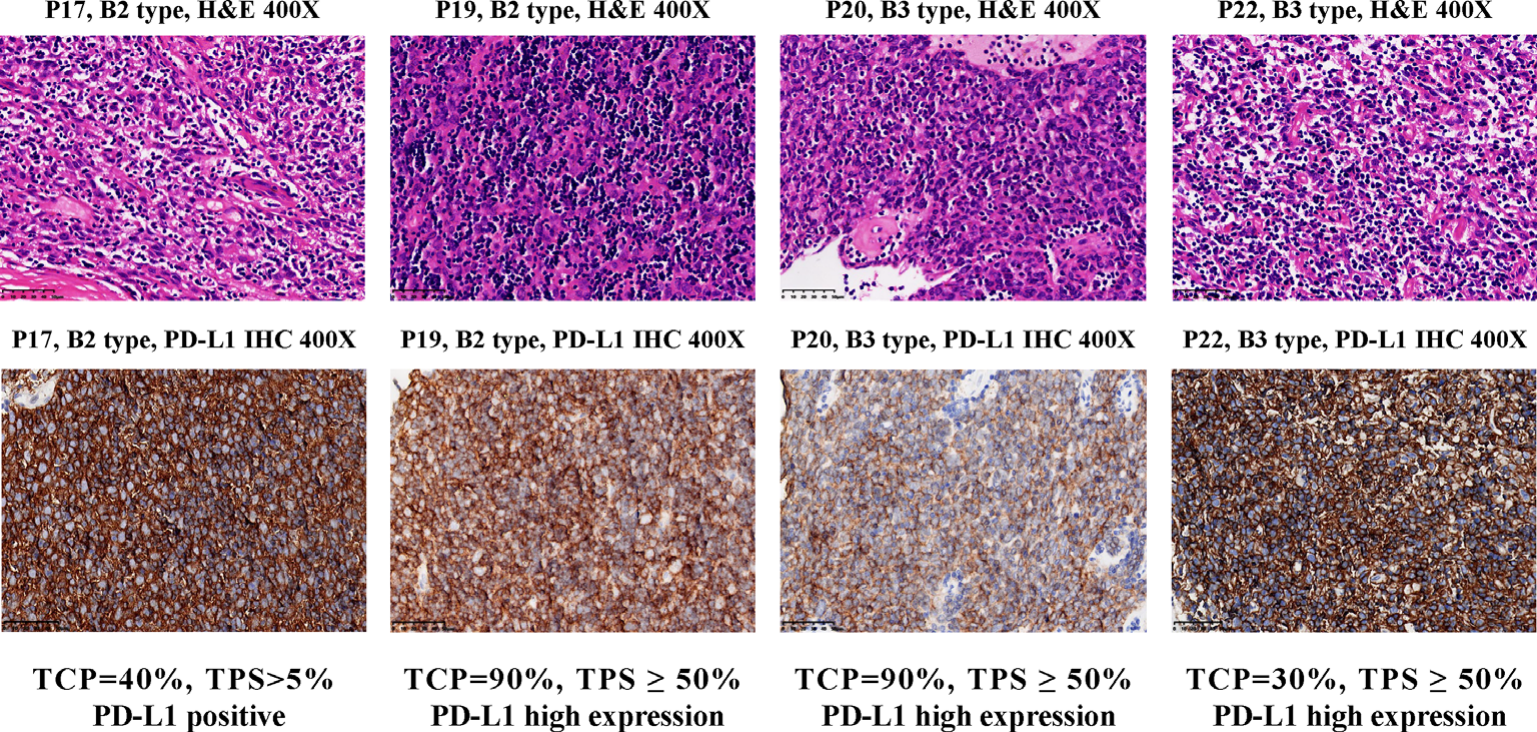
PD-L1 IHC staining for B2 and B3 samples in the cluster C4. Top, hematoxylin and eosin (H&E) staining of the tissues. Bottom, IHC staining of PD-L1 in the cells using antibody 22C3 (Dako). Brown cells are PD-L1 staining cells. The H&E staining was used to estimate the tumor cell percentage (TCP). The expression level of PD-L1 protein is determined by the tumor proportion score (TPS), which is further divided into three types: ‘High expression’ (TPS ≥ 50%), ‘Positive’ (1% ≤ TPS < 50%) and ‘Negative’ (TPS < 1%).

C4 consisted of five B2 or B3 subtypes. *FGF10*, a regulator of cortex epithelial cell proliferation, was highly expressed in C4 cluster (**Figures 3E** and **S5E**), which may be related to the mechanism of self-maintenance of proliferation of B2 and B3 subtypes. Importantly, *CD274* (PD-L1, **Figures 3F** and **S5F**) and *PDCD1LG2* (PD-L2, **Figure S7A**), encoding PD-1 ligand, were highly expressed in this cluster. We further performed PD-L1 IHC and the results showed the patients with B2 or B3 thymomas in the cluster C4 had high PD-L1 expressions (**Figure 4**), which suggested patients with B2 and B3 thymomas could potentially benefit from immunotherapy (35–37). The biological roles for the highly expressed genes in C5 remain to be further investigated.

Expression profiling and pathway analysis showed the differences of highly expressed genes in each cluster. These data showed that histologic subtypes and molecular clustering patterns were mostly consistent, which was also supported by the TCGA analysis. For low-risk subtypes (A and AB), the molecular function was associated with tissue development and cell proliferation. The absence of transcription factors, such as *EHF* and *E2F8*, may represent the initial steps of gene dysregulation. For the more malignant types (B2 and B3, and TC), more cancer progression related genes were highly expressed. Several important genes associated with thymus development, such as *CLDN4*, *FGF7* and *FGF10*, showed high expression in certain thymoma subtypes and TCs, suggesting their potential role in the development of TETs.

### 3.7 Myasthenia Gravis Related Gene Analysis on Thymic Epithelial Cancer

Thymoma is often associated with an autoimmune thymus disease MG. We found 149 genes with differential expression between MG+ and MG- groups (**Supplementary Table 6**). Nonetheless, these DEGs did not contain genes involved in immunity or auto-antigens. Among the auto-antigen related DEGs between MG+ and MG- reported in TCGA, *NEFM* was highly expressed in the MG+ group in our cohort, but there was no statistically significant difference in comparison with the MG- group (**Figure S9**).

To investigate whether the MG status can be inferred from the gene expression profiles of TET patients, we constructed a machine learning model using SVM, in which the TCGA-TET samples (n = 104) were used as the training set to select the gene features, and 22 samples in our cohort were used as a validation set to verify the prediction performance of the model. We found that a three-gene model had optimal prediction performance between the TCGA cohort and our cohort (**Figure S10**). The AUC of the training and validation set was comparable (0.840 vs 0.841), with 93.8% sensitivity and 64.0% specificity for the training set, and 90% sensitivity and 70.6% specificity in the testing cohort. The three genes used in this model were *PPARGC1A*, *GABRA5*, and *NEFM*, in which *NEFM* was related to MG according to the previous reports and the TCGA TET study (6).

## 4 Discussion

The rare occurrence of TETs hinders clinical research and biomarker discovery. The recent integrative multi-omics study extended the knowledge of molecular signatures to the clinical characteristics of thymic epithelial tumors (6). In this pilot study, we performed genomic and transcriptomic analysis to explore the underlying molecular characteristics and mechanisms of a Chinese TET cohort. To the best of our knowledge, this is the first multi-omics study reported on a Chinese TET population.

The mutational landscape of our cohort showed several key differences from the TCGA dataset. First, the top mutated genes in our cohort did not match those in the TCGA cohort. Prevalent mutations in *NF1* and *ATM* suggest potential difference of the mutational landscape in the Chinese TET population. Though our sample size was small, the difference in the mutation frequency of *GTF2I* L424H on the B subtypes still can provide preliminary evidence that the Chinese TET population may have different mutational landscape compared with the TCGA dataset. Another example of different mutational profiles between Chinese and Caucasians is different prevalence of driver mutations in lung adenocarcinoma (LUAD) (38). While east Asian LUAD patients are frequently mutated on *EGFR* L858R and 19del (40-55%) and less occurrence of *KRAS* mutations (8-12%), the Caucasians have a lower incidence of *EGFR* mutations (15-25%) and higher incidence of *KRAS* mutations (20-30%). The etiology of the Chinese TET patient could be possibly different from Caucasian population represented in TCGA. However, the expression profiles showed no obvious differences between different population groups, and the myasthenia gravis prediction model worked well for both cohorts. These results suggest that mutational landscape, which may involve in tumorigenesis, differs in populations, but with overall consistent expression patterns among them.

Consistent with the TCGA’s findings, expression profiling clusters was largely consistent with the pathological subtypes. We further demonstrated that the less malignant TETs had more gene expression of developmental processes and cellular components whereas the more malignant TETs had more genes with altered expression that associated with immune system and cell adhesion/migration. It was interesting to point out that several genes related to thymus medullary and cortex development such as *CLDN4*, *FGF7*, and *FGF10* were linked to TETs. Such relationships unveil possible links between tumorigenesis and dysregulation of regulatory networks that can lead to an insightful understanding of the etiology of the disease. Targeting those genes might provide potential therapeutic clues with further cellular mechanism and clinical studies.

The expression profiling analysis also identified high expression of PD-L1 for most of the B2 and B3 subtypes in our cohort, suggesting immunotherapy opportunity for those patients. This is consistent with the previous studies which reported high PD-L1 expression in TET patients with more malignant subtypes, such as type B2 and B3 thymomas, and thymic carcinomas (39, 40). Moreover, we proposed here the first machine learning model to predict myasthenia gravis status for TET patients, which provides new perspectives for tackling the problem and could probably help high risk myasthenia gravis patients in advance.

The limitation of this study is the relatively small number of cases in view of the heterogeneity of histological subtypes in TET patients. The size of the sample was largely restricted by the low incidence of TET and the sample availability from TET patients. Another limitation is that we applied targeted gene panel rather than whole exome or whole-genome sequencing. Though the panel is relatively large and contains 476 closely cancer related genes, the resulting mutation profiles may still be limited and possibly miss novel somatic mutations associated with Chinese TET patients. Future studies should expand the sample size to further validate both the molecular profiles and the MG prediction model in Chinese TET patients and clinical applications.

## Data Availability Statement

The original contributions presented in the study are included in the article/**Supplementary Material**. Further inquiries can be directed to the corresponding authors.

## Ethics Statement

The studies involving human participants were reviewed and approved by Ethics Committee of the Peking Union Medical College Hospital. The patients/participants provided their written informed consent to participate in this study.

## Author Contributions

All authors contributed to the article and approved the submitted version. Tissue experiments were performed by NLiang, LL, JL, CH, HL, CG, WW, and RL. Pathological diagnosis was conducted by JL. Data analysis were performed by NLi, and TW. The article was written by NLiang, NLi, RL, TW, LM, and SL. Funding was acquired by NLiang and SL.

## Funding

This work was supported by the Foundation for Key Program of Ministry of Education, China (Grant No. 311037), CAMS Innovation Fund for Medical Sciences (2017-12M-1-009), and Beijing Natural Science Foundation (7182132). 

## Conflict of Interest

NLi, RL, and TW are employees at Repugene Technology. LD and LM are employees and shareholders at Betta Pharmaceuticals.

The remaining authors declare that the research was conducted in the absence of any commercial or financial relationships that could be construed as a potential conflict of interest.

## Publisher’s Note

All claims expressed in this article are solely those of the authors and do not necessarily represent those of their affiliated organizations, or those of the publisher, the editors and the reviewers. Any product that may be evaluated in this article, or claim that may be made by its manufacturer, is not guaranteed or endorsed by the publisher.
