# Supplementary material for: Transcriptomic and Mutational Analysis Discovering Distinct Molecular Characteristics Among Chinese Thymic Epithelial Tumor Patients
Source: Front Oncol. 2021 Sep 8;11:647512. doi: 10.3389/fonc.2021.647512 (PMC8456088; doi:10.3389/fonc.2021.647512)
Supplement: Supplementary file 1 [file DataSheet_1.docx]

**Supplementary Figures S1-S10**

**Transcriptomic and mutational analysis discovering distinct molecular characteristics among Chinese thymic epithelial tumor patients**

Naixin Liang^1#^, Lei Liu^1#^, Ji Li^2^, Cheng Huang^1^, Hongsheng Liu^1^, Chao Guo^1^, Weiwei Wang^1^, Nan Li^3^, Rui Lin^3^, Tao Wang^3^, Lieming Ding^4^, Li Mao^4*^, Shanqing Li^1*^

1. Department of Thoracic Surgery, Peking Union Medical College Hospital, Chinese Academy of Medical Sciences, Beijing 100730, China

2. Department of Pathology, Peking Union Medical College Hospital, Chinese Academy of Medical Sciences, Beijing 100730, China

3. Hangzhou Repugene Technology Co., Ltd., Hangzhou 311100, China

4. Betta Pharmaceuticals Co., Ltd., Hangzhou 311100, China

# The authors contribute equally to the manuscript


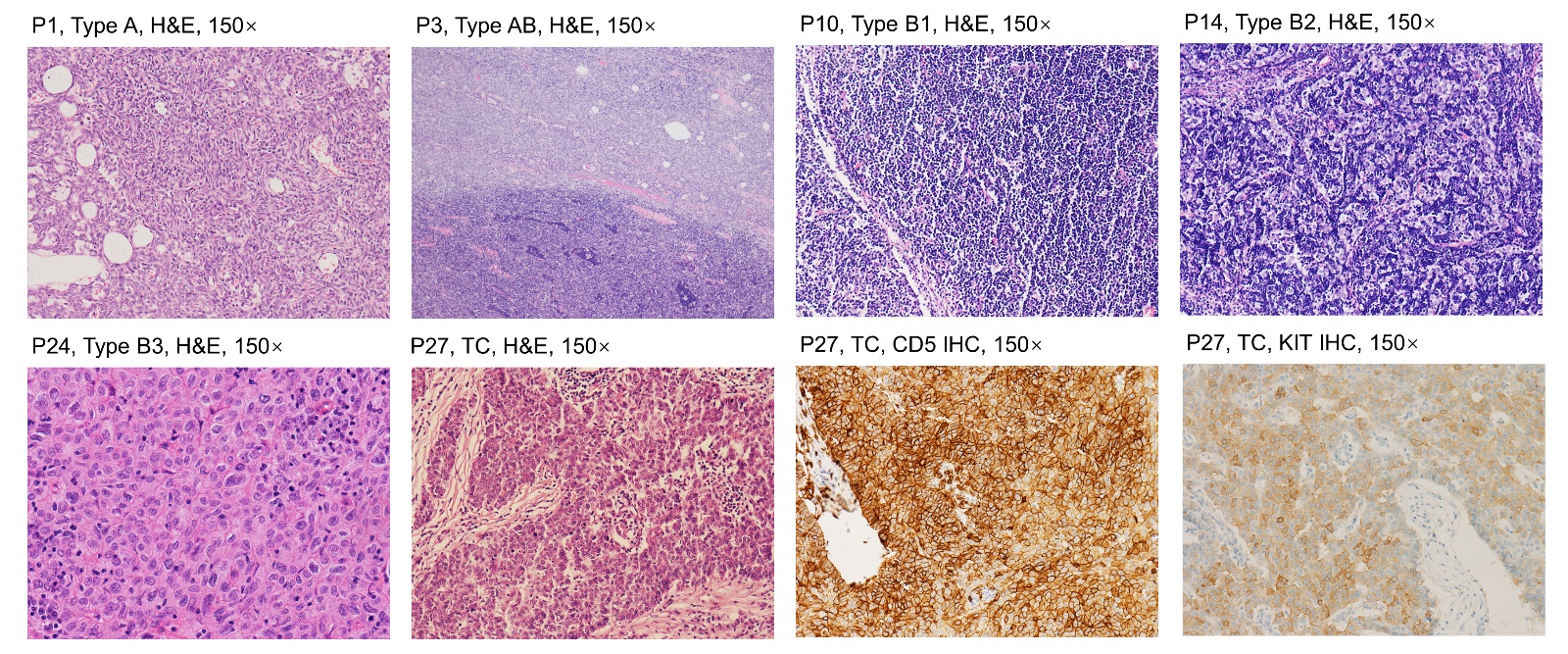


**Figure S1**. The representative hematoxylin and eosin (H&E) and immunohistochemistry (IHC) staining results for each subtype (A, AB, B1, B2, B3 and TC) of TET.

**
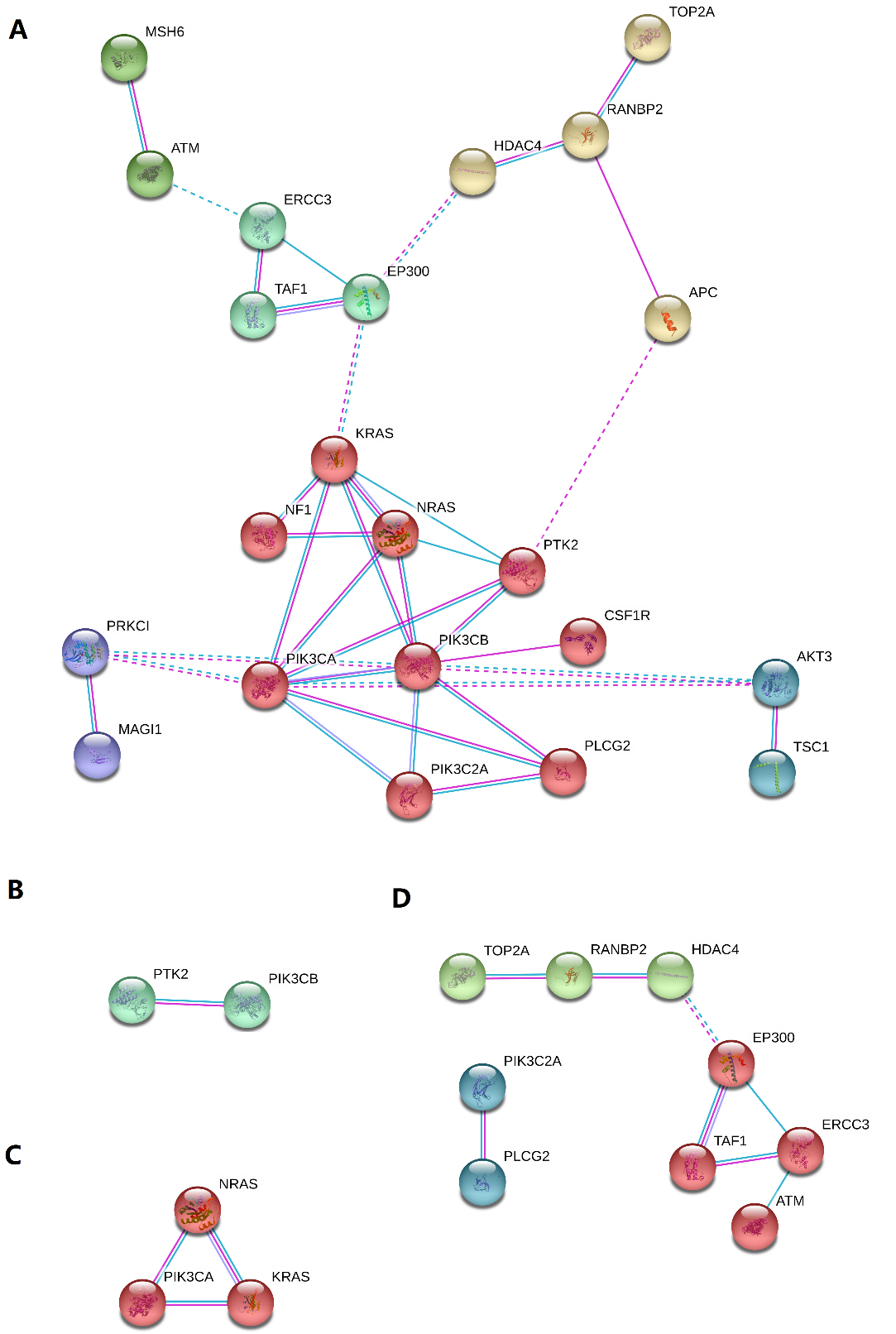
**

**Figure S2.** Protein-protein interaction clustering of mutated genes. Color shows the cluster assignment of each gene. The blue and pink edge indicate that the interaction comes from the interaction database and experimental validation respectively. Solid edges show intra-cluster connection and dot edges inter-cluster connections. **A** Network of mutated genes for all samples. **B** Network of mutated genes from A type samples. **C** Network of mutated genes from AB type samples. **D** Network of mutated genes from B type samples.


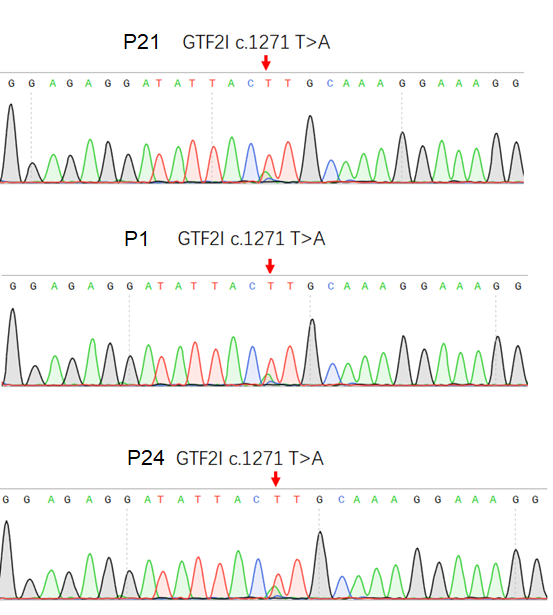


**Figure S3.** Sanger sequencing validation of *GTF2I* c.1271T>A (p.L424H) on three representative samples. Arrow indicates the site of mutation.


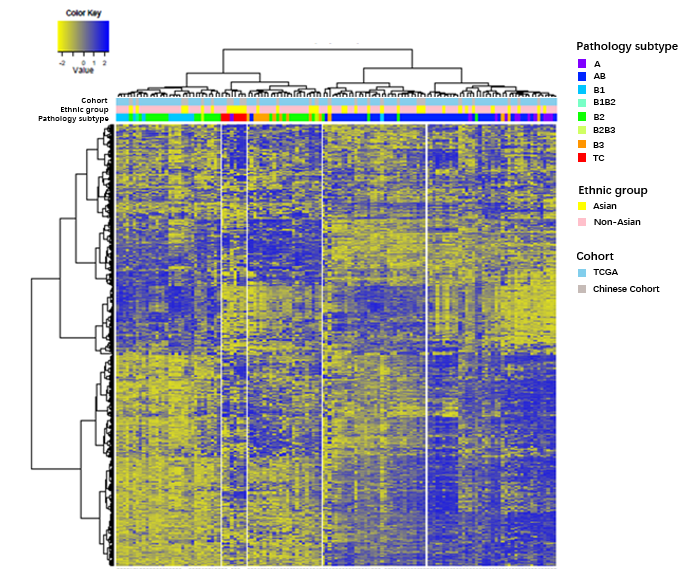


**Figure S4.** Hierarchical clustering of combined RNA-seq data from samples in the current study and from the TCGA cohort.
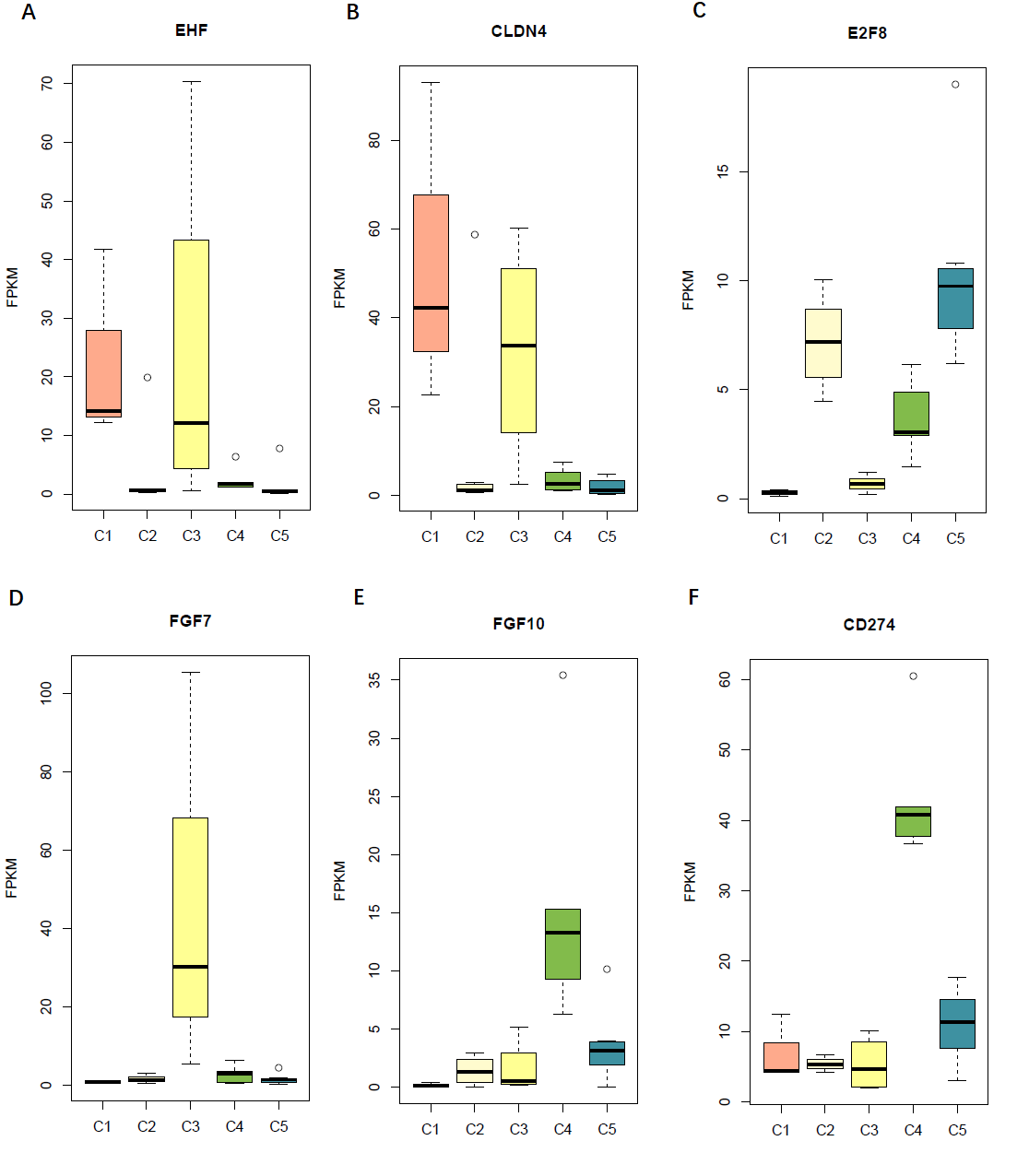


**Figure S5.** Gene expression boxplots of five clusters for genes in Figure 3. The match of pathological subtype and RNA-seq clusters are: A-C1, AB-C2, TC-C3, B2/B3-C4, and B1/B2-C5.


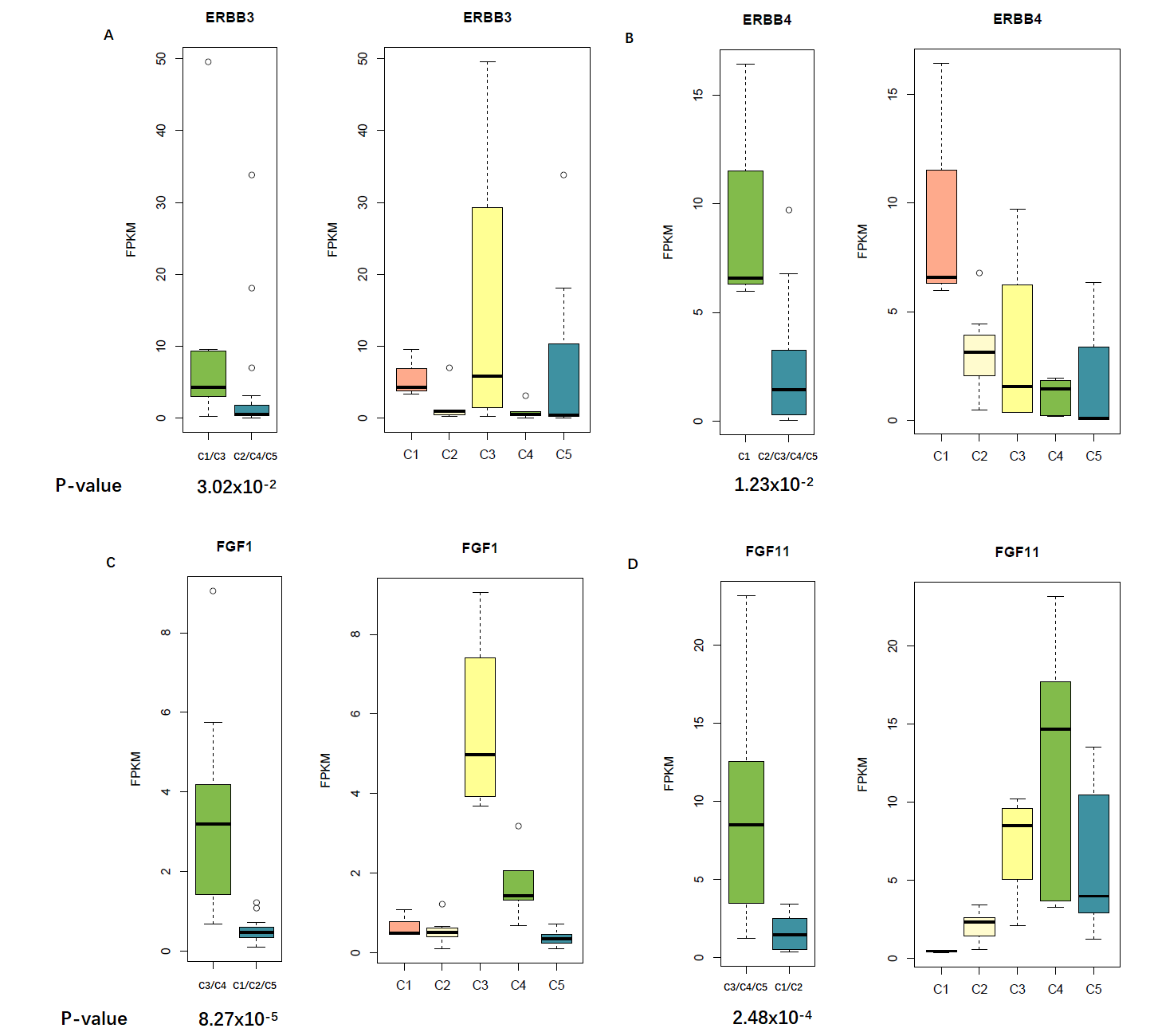


**Figure S6.** The expression boxplots of ERBB3, ERBB4, FGF1, and FGF11. The match of pathological subtype and RNA-seq clusters are: A-C1, AB-C2, TC-C3, B2/B3-C4, and B1/B2-C5.


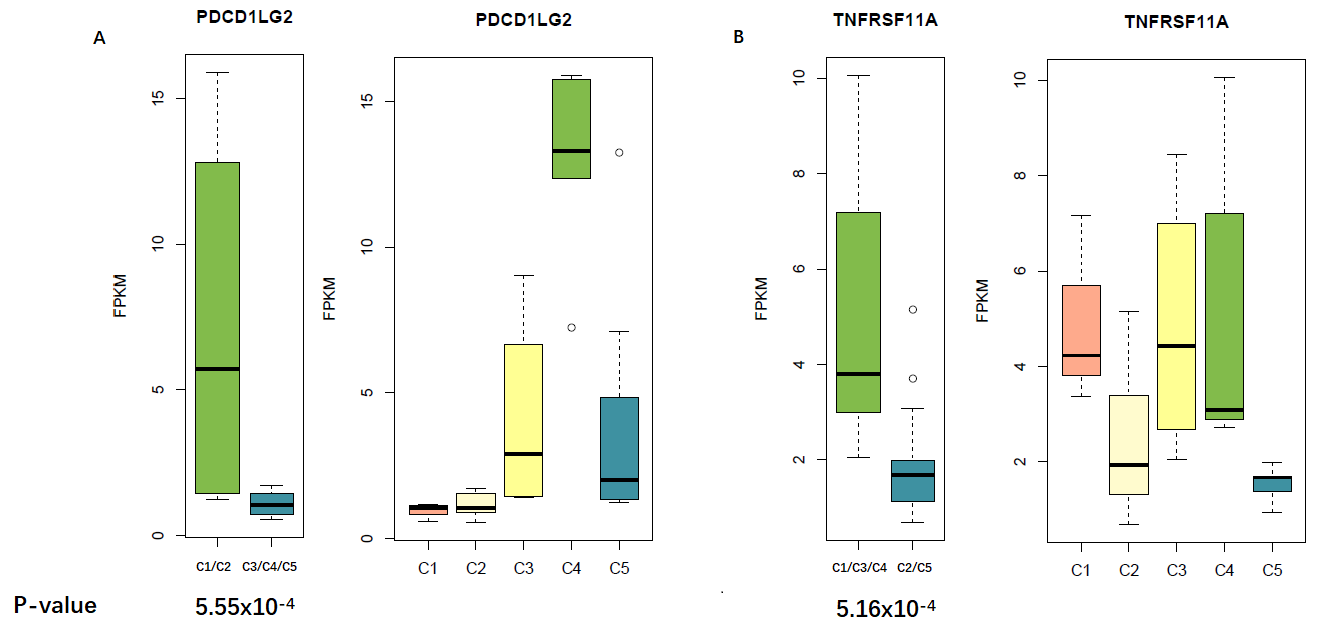


**Figure S7.** The expression boxplots of PDCD1LG2 and TNFRSF11A. The match of pathological subtype and RNA-seq clusters are: A-C1, AB-C2, TC-C3, B2/B3-C4, and B1/B2-C5.


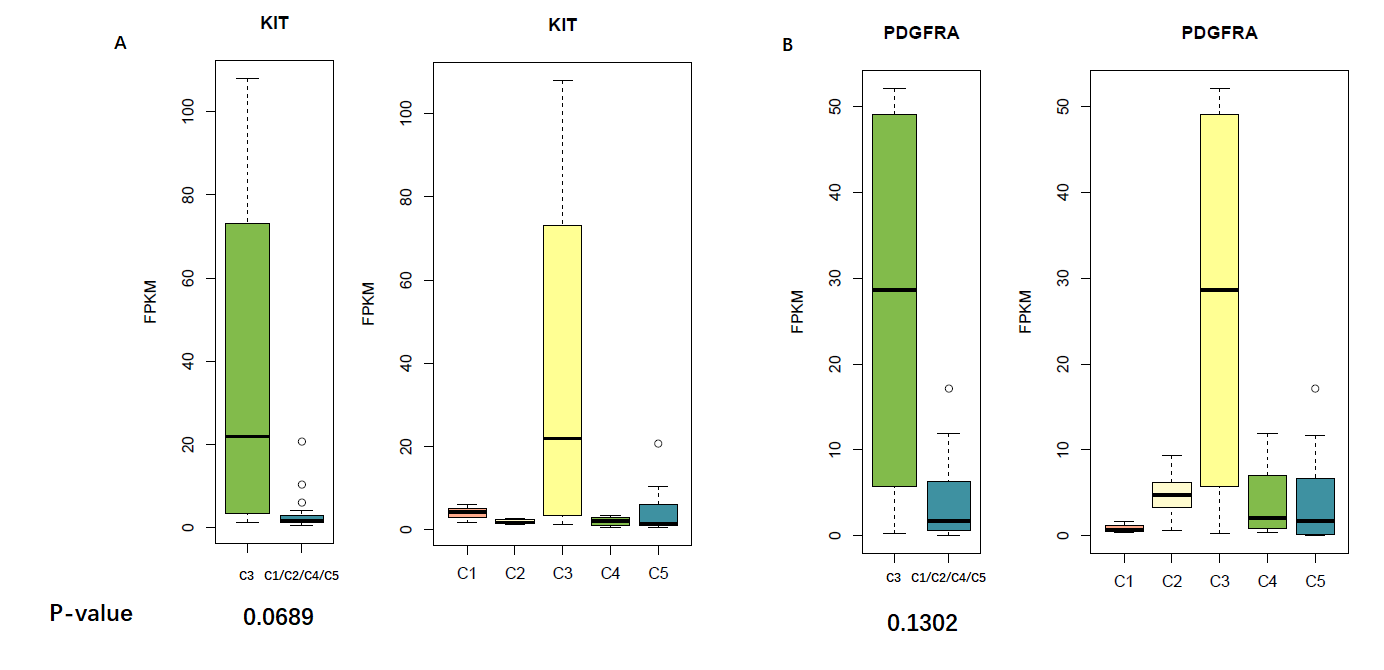


**Figure S8.** The expression boxplots of KIT and PDGFRA. The match of pathological subtype and RNA-seq clusters are: A-C1, AB-C2, TC-C3, B2/B3-C4, and B1/B2-C5. FPKM, fragment per kilo exon per million reads.


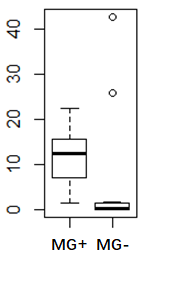


**Figure S9.** Expression boxplot of NEFM in MG+ and MG- groups. Y axis, FPKM (fragment per kilo exon per million reads) value; MG, myasthenia gravis.


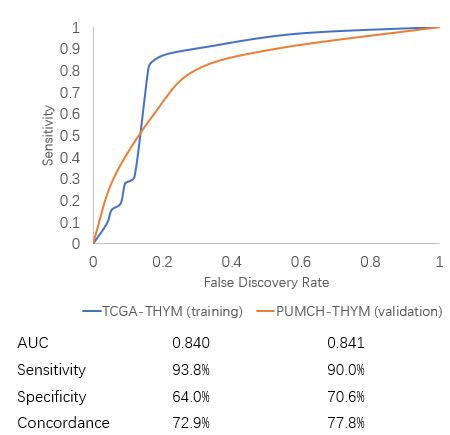


**Figure S10.** Prediction of MG status on thymoma samples using support vector machine (SVM) model. TCGA-THYM, the cohort from TCGA study; PUMCH-THYM, the cohort from the current study; AUC, area under the curve.
