# Supplementary material for: Transcriptomic and Mutational Analysis Discovering Distinct Molecular Characteristics Among Chinese Thymic Epithelial Tumor Patients
Source: Front Oncol. 2021 Sep 8;11:647512. doi: 10.3389/fonc.2021.647512 (PMC8456088; doi:10.3389/fonc.2021.647512)
Supplement: Supplementary file 3 [file Table_2.docx]

**Table S2**. Representative computed tomography (CT) scans of the thymus of each subtype of TET.

| **Patient ID** | **Histological Class** | **Masaoka Stage** | **TNM Stage** | **CT scan** |
| --- | --- | --- | --- | --- |
| P1 | A | I | T1N0M0, I | 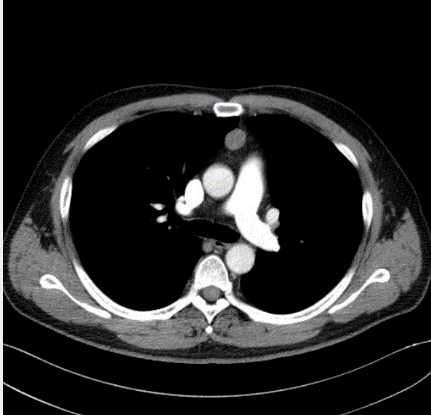 |
| P3 | AB | I | T1N0M0, I | 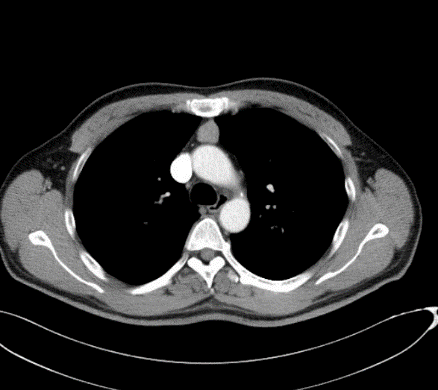 |
| P9 | B1 | I | T1N0M0, I | 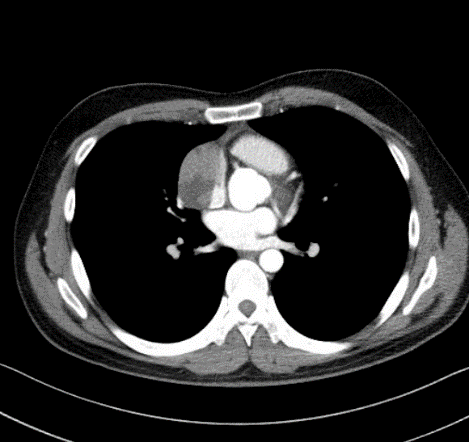 |
| P18 | B2 | II | T2N0M0, II | 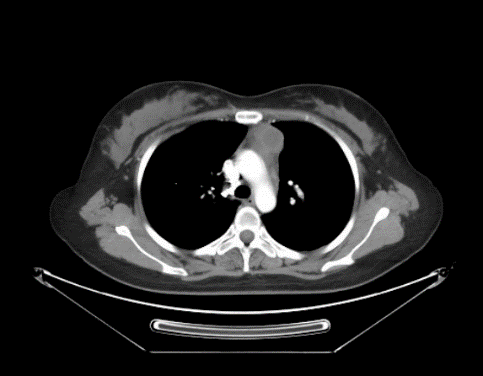 |
| P20 | B3 | III | T3N0M0, IIIA | 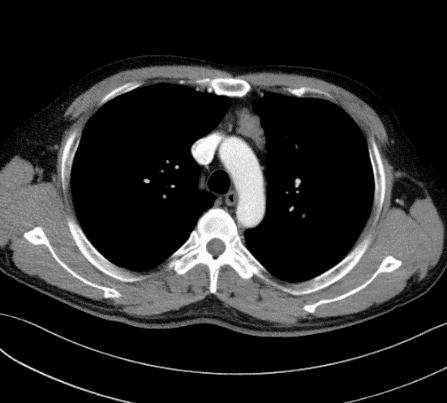 |
| P27 | TC (squamous carcinoma) | III | T3N0M0, IIIA | 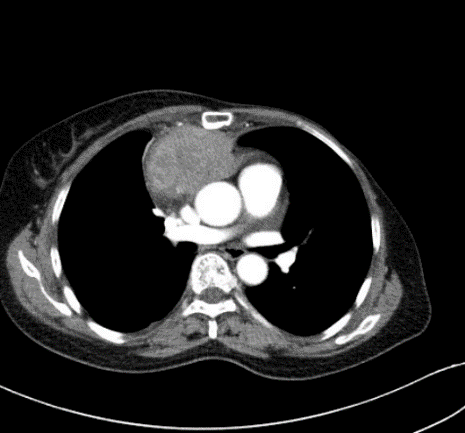 |
